# Identification of a nonsense mutation in *TNNI3K* associated with cardiac conduction disease

**DOI:** 10.1002/jcla.23418

**Published:** 2020-06-11

**Authors:** Jiang Liu, Da Liu, Muzheng Li, Keke Wu, Na Liu, Chenyu Zhao, Xiaoliu Shi, Qiming Liu

**Affiliations:** ^1^ Department of Cardiology The Second Xiangya Hospital of Central South University Changsha China; ^2^ Department of Gastroenterology The Second Xiangya Hospital of Central South University Changsha China; ^3^ Department of Medical Genetics The Second Xiangya Hospital of Central South University Changsha China

**Keywords:** cardiac conduction disease, nonsense mutation, nonsense‐mediated mRNA decay, *TNNI3K*, whole‐exome sequencing

## Abstract

**Background:**

Cardiac conduction disease (CCD) is a common cardiovascular disease which can lead to life‐threatening conditions. The importance of heredity in CCD has been realized in recent years. Several causal genes have been found to be implicated in CCD such as *SCN5A*, *TRPM4*, *SCN1B*, *TNNI3K*, *LMNA, and NKX2.5*. To date, only four genetic mutations in *TNNI3K* have been identified related to CCD.

**Methods:**

Whole‐exome sequencing (WES) was carried out in order to identify the underlying disease‐causing mutation in a Chinese family with CCD. The potential mutations were confirmed by Sanger sequencing. Real‐time qPCR was used to detect the level of *TNNI3K* mRNA expression.

**Results:**

A nonsense mutation in *TNNI3K* (NM_015978.2: g.170891C > T, c.1441C > T) was identified in this family and validated by Sanger sequencing. Real‐time qPCR confirmed that the level of *TNNI3K* mRNA expression was decreased compared with the controls.

**Conclusions:**

This study found the first nonsense *TNNI3K* mutation associated with CCD in a Chinese family. *TNNI3K* harboring the mutation (c.1441C > T) implicated a loss‐of‐function pathogenic mechanism with an autosomal dominant inheritance pattern. This research enriches the phenotypic spectrum of *TNNI3K* mutations, casting a new light upon the genotype‐phenotype correlations between *TNNI3K* mutations and CCD and indicating the importance of *TNNI3K* screening in CCD patients.

## 1 INTRODUCTION

Cardiac conduction disease (CCD) refers to the impaired completeness of conduction system which can be serious and potentially life‐threatening. Based on part upon the site of conduction block, CCD is recognized, namely sick sinus syndrome, intra‐atrial block, atrioventricular block, and intraventricular conduction delay. More than 20 genes have been implicated in harboring rare variants that cause CCD, containing genes that encode ion channel, transcriptional factors, enzymes, and structural proteins such as *SCN5A* (OMIM#600163; 3p22.2), *TRPM4* (OMIM#606936; 19q13.33), *NKX2.5* (OMIM#600584; 5q35.1), *TBX5* (OMIM#601620; 12q24.21), *PRKAG2* (OMIM#602743; 7q36.1), and *LMNA* (OMIM#150330; 1q22).^1^, ^2^ CCD continues to be the major cause for pacemaker (PM) implantation irrespective of its diverse underlying pathophysiological mechanisms.^3^, ^4^

*TNNI3K* (OMIM #613932), located in chromosome 1 (1p31.1), encodes a dual‐function kinase (both tyrosine and serine/threonine kinase activity) with biased expression in heart.^5^, ^6^ It contains four domains as follows: an N‐terminal coiled‐coil domain, ankyrin (ANK) repeats, protein kinase domain, and a C‐terminal serine‐rich domain. Interaction partners of *TNNI3K* embody cardiac troponin I (cTnI), anti‐oxidant protein 1 (AOP‐1), and p38, which make *TNNI3K* an important factor in cardiovascular diseases.^7^, ^8^ *TNNI3K* was related to heart failure and hypertrophy, ischemia/reperfusion injury, cardiac conduction, and heart regeneration.^9^, ^10^, ^11^, ^12^, ^13^, ^14^, ^15^ Rare mutations related to cardiovascular diseases have been identified in more than 100 genes which encode proteins belonging to different cellular structures and pathways such as cytoskeleton, sarcomere, nuclear membrane, ion channel, mitochondria, sarcoplasmic reticulum, and desmosomes. To date, there are only four mutations in *TNNI3K* that have been found to be relevant with cardiovascular diseases, including three missense mutations and one splicing mutation.^16^, ^17^, ^18^, ^19^ Here, we found that a nonsense variant in *TNNI3K* (NM_015978.2:c.1441C > T), which was not seen in the human gene mutation database (HGMD), may be the cause of cardiac abnormalities.

## 2 MATERIALS AND METHODS

### 2.1 Patients enrollment and ethical approval of the study

**Figure 1:**
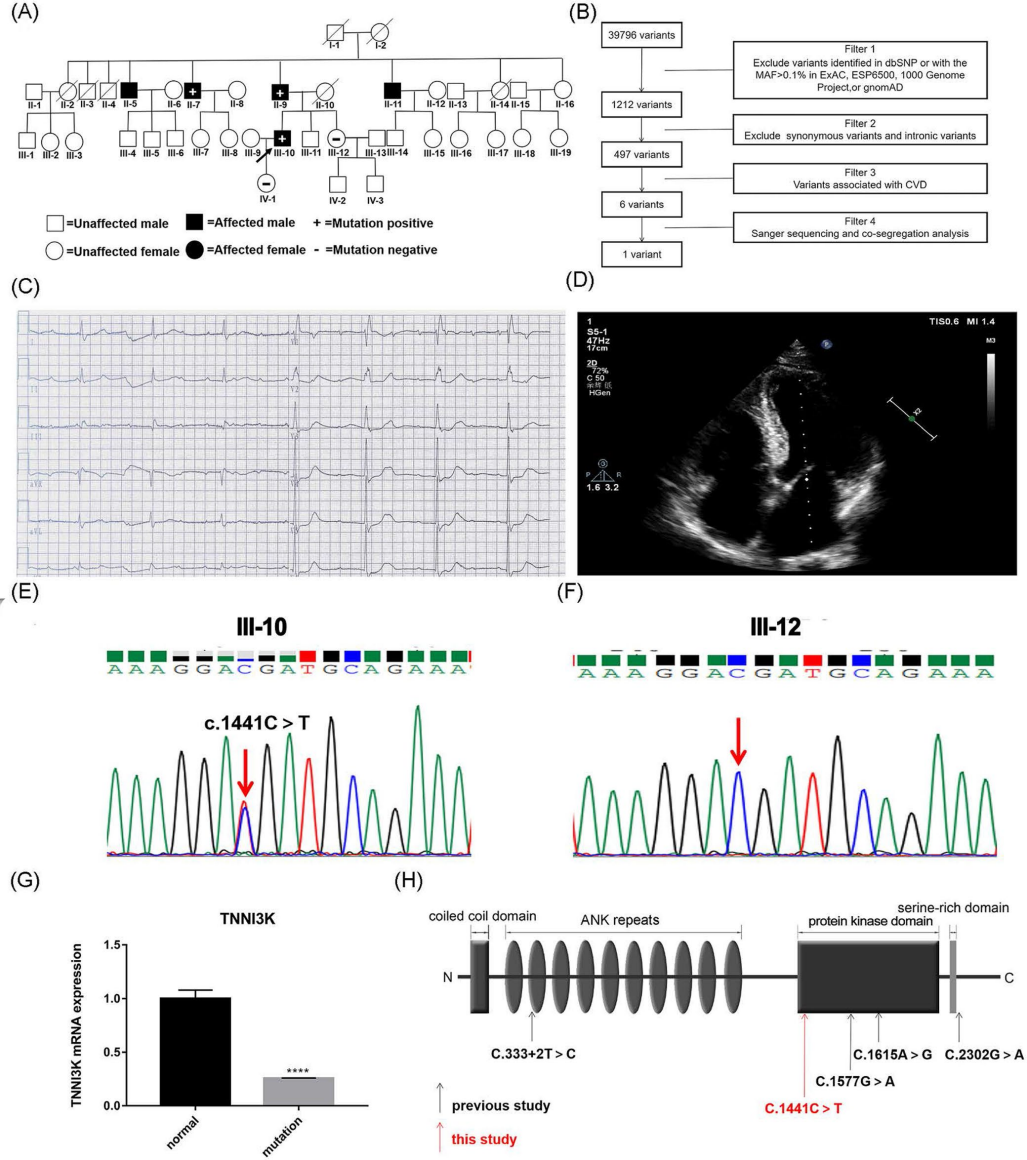
The clinical data and genetic analysis in our study. A, Pedigree. *TNNI3K* c.1441C > T variant was positive in individuals (II‐7, II‐9, and III‐10) and negative in individuals (III‐12 and IV‐1). B, Filter strategies in our research. C, The ECG record of the proband (III‐10) before PM implantation. D, The echocardiography of III‐10. E‐F, Sanger sequencing confirmation of III‐10 and III‐12.G, RNA expression of *TNNI3K* in affected individuals and controls. Mean expression (±SEM) of *TNNI3K* in affected individual and control measured by real‐time qPCR.**** represents *P* < .0001. H, Mapping of the domain with *TNNI3K* variants identified in previous studies (denoted by black arrows) and this study (denoted by red arrow). ANK, ankyrin; CVD, cardiovascular diseases; ECG, electrocardiogram; PM, pacemaker

A Chinese family with cardiovascular diseases was enrolled in our studies. Parents of the proband are not related biologically. The pedigree of the family is shown in Figure 1A. Written informed consent was obtained from each individual, and the investigation was approved by the Ethics Committee of The Second Xiangya Hospital of Central South University.

### 2.2 DNA extraction and whole‐exome sequencing

DNA was extracted from blood samples of the proband (III‐10) and family members (II‐7, II‐9, III‐12, and IV‐1). Other members' blood samples were not available. Whole‐exome sequencing (WES) was performed on the proband. WES and basic bioinformatics analyses were finished in the Novogene Bioinformatics Institute (Beijing, China). Platform for high‐throughput sequencing was Illumina novaSeq. The reads data were mapped to the human reference genome 19 (UCSC hg19) using BWA. Single‐nucleotide variants (SNVs) and insertion‐deletion variants (INDELs) were annotated by ANNOVAR. Low‐quality data (cover depth＜20×) and high‐frequency mutation sites (minor allele frequency [MAF] >0.001 in ExAC, ESP6500,1000 Genome Project, and gnomAD) in general population were eliminated from raw data. SIFT, PolyPhen‐2, MutationTaster, and PROVEAN were used for predicting pathogenicity of mutations. Interpretation of mutations pathogenicity was guided by American College of Medical Genetics and Genomics guideline (ACMG).^20^ The filter strategies are shown in Figure 1B and Table 1. The validation of potential mutations in the proband and his family members was done by means of Sanger sequencing. Primer 3 was used to design the primer pairs (primers Tm and sequences that have been used in co‐segregation were showed in Table. S1). ABI 3730 sequencer (Applied Biosystems) was used for Sanger sequencing.

**Table 1:** The gene list of Sanger sequencing validation and co‐segregation analysis in this family

Gene	OMIM	CHR	POS	RB	AB	AAChange	SIFT	PolyPhen‐2	Mutationtaster
TNNI3K	613932	1p31.1	74834917	C	T	NM_015978:exon15:c.C1441T	—	—	1.000, D
RYR2	180902	1q43	237947718	T	G	NM_001035:exon90:c.T12706G	0.5, T	0.257, B	1.000, D
TTN	188840	2q31.2	179425543	C	T	NM_001267550:exon326:c.G85316A	0, D	0.701, P	1.000, D
CACNA1C	114205	12p13.33	2788847	C	T	NM_000719:exon42:c.C5329T	0.002, D	0.999, D	1.000, D
NUBPL	613621	14q12	32319403	A	C	NM_001201573:exon8:c.A605C	0.23, T	0.002, B	0.764, N
MYOM1	603508	18p11.31	3164344	C	A	NM_003803:exon10:c.G1433T	0.31, T	1.000, D	0.764, N

Abbreviations: AB, alternative base identified; B, benign; CHR, chromosome; D, disease‐causing; N, neural; POS, position; RB, reference sequencing base; T, tolerated.

### 2.3 Real‐time qPCR analysis

Total RNA was extracted by the PureLink^®^ RNA Mini (Thermo Fisher Scientific; #12183025) from mononuclear cells in the affected patients and healthy controls. cDNA was synthesized from a total of 1 μg of RNA using the RevertAid First Strand cDNA Synthesis Kit (Thermo Fisher Scientific; #K1621) with oligo (dT) primers. Real‐time qPCRs were carried out in Fast 7500 Real‐Time PCR Systems (Applied Biosystems) using Maxima SYBR Green/ROX qPCR Master Mix (2×) (Thermo Fisher Scientific, #K0221). And 2 (−^△△^ *^C^* ^t^) was used to anal

yze the comparative *TNNI3K* mRNA expression levels between mutation group and healthy group. Each assay was performed in five independent tests. The data were analyzed by unpaired two‐tailed tests using GraphPad Prism V.5 software (V.5.0). The primers used for cloning *TNNI3K* cDNA were as follows: forward 5′‐CTAGAGGCTGCTGATGTGCTGTTG‐3′; reverse 5′‐GGCGAGTTACCTGTTCATGTCCATAG‐3′.

## 3 RESULTS

### 3.1 Clinical data

The index case (III‐10) is a 56‐year‐old man from China with the clinical manifestation of chest tightness. The electrocardiogram (ECG) showed complete right bundle branch block (CRBBB)and third‐degree atrioventricular block (Figure 1C). The 24‐hour Holter indicated an average heartbeat of 46 bpm, maximum RR interval of 5.26 seconds, complete atrioventricular block, and CRBBB. His Echocardiography displayed enlargement of left atrial (LA = 40 mm), thickened left ventricular posterior wall (LVPW = 13 mm), and interventricular septum (IVS = 14 mm; Figure 1D). His father and uncles (II‐9, II‐5, II‐7, and II‐11) were also suffered from third‐degree atrioventricular block and were treated with PM therapy among their 50s. Results of echocardiography were all normal before their PM implantation. Cardiac enlargement of his uncles (II‐5 and II‐7) emerged after a period of PM implantation. The proband's aunt (II‐14) died of heart disease at her 30s recalled by him (no more details available). Owing to the implantation of PM, cardiac magnetic resonance is not performed in this family (Table 2 and Figure S1A‐D).

**Table 2:** Clinical characteristics and phenotype of family members

Subject	Sex	Age (y/old)	Age of PM Implantation (y/old)	UCG	Manifestation
III‐10 Proband	M	56	56	LA = 40 mm, LV = 46 mm, RA = 35 mm, RV = 34 mm, IVS = 14 mm, LVPW = 13 mm, EF = 65%	3rd‐degree AVB, RBBB, PVC
II‐5 Uncle	M	91	55	LA = 38 mm, LV = 65 mm, RA = 37 mm, RV = 37 mm, EF = 55%	3rd‐degree AVB, PVC, AT
II‐7 Uncle	M	89	54	LA = 42 mm, LV = 62 mm, RA = 37 mm, RV = 39 mm, EF = 40%	3rd‐degree AVB, PVC, RBBB
II‐9 Father	M	86	59	LA = 38 mm, LV = 50 mm, RA = 34 mm, RV = 35 mm, EF = 54%	3rd‐degree AVB, PVC
II‐11 Uncle	M	84	55	LA = 33 mm, LV = 49 mm, RA = 30 mm, RV = 34 mm, EF = 60%	3rd‐degree AVB, RBBB
III‐12 Sister	F	52	/	LA = 29 mm, LV = 44 mm, RA = 28 mm, RV = 26 mm, EF = 66%	/
IV‐1 Daughter	F	26	/	LA = 27 mm, LV = 43 mm, RA = 27 mm, RV = 28 mm, EF = 66%	/

Abbreviations: AT, atrial tachycardia; AVB, atrioventricular block; EF, ejection fraction; F, female; IVS, interventricular septum; LA, left atrial; LV, left ventricle; LVPW, left ventricular posterior wall; M, male; PM, pacemaker; PVC, premature ventricular contraction; RA, right atrial; RBBB, right bundle branch block; RV, right ventricle; UCG, ultrasonic cardiography.

### 3.2 Genetic testing

A nonsense mutation in *TNNI3K* (c.1441C > T, p.R481X) was found through WES and confirmed by Sanger sequencing (Figure 1E‐F and Figure S1E‐F). This variant produces a truncated protein with 480 amino acids which is significantly shorter than the wild‐type protein (835 amino acids). It is a rare mutation with MAF of 0.0017%(2/119514 alleles) in ExAC database. In addition, this mutation was not seen in our 200 local control cohorts.^21^ The c.1441C > T mutation in the *TNNI3K* gene co‐segregated with the clinical phenotype in this family. Thus, we believed that this mutation seems to be the causative mutation of cardiac abnormalities in this family.

### 3.3 Real‐time qPCR analysis

The nonsense mutation results in the early appearance of stop codon which is anticipated to activate a process called nonsense‐mediated mRNA decay (NMD).^22^ We isolated the mRNA from the mononuclear cells in the patients and healthy family members. Real‐time qPCR regarded the healthy control levels of mRNA in *TNNI3K* as “1.” The results revealed a decreased level of *TNNI3K* mRNA expression in patients compared with the controls (*P* < .0001; Figure 1G).

## 4 DISCUSSION

Herein, using WES we have identified a nonsense mutation of the *TNNI3K* gene associated with CCD. Cardiovascular disease caused by the *TNNI3K* mutation is an autosomal dominant disease, which means individuals will have the disease if only one chromosome carries the mutant allele. In our study, the nonsense mutation (c.1441C > T) is co‐segregated with the affected ones. The real‐time qPCR test revealed that the expression level of *TNNI3K* mRNA in affected members was decreased compared with healthy controls. Our study is consistent with previous studies which demonstrated that *TNNI3K* mutations were related to CCD with or without dilated cardiomyopathy.

The gene *TNNI3K* holds a place in cardiac physiology. A preponderance of evidence suggested that *TNNI3K* is associated with a broad spectrum of cardiac phenotypes including CCD, dilated cardiomyopathy, and supraventricular tachycardia. Only four disease‐causing mutations in *TNNI3K*, namely three missense mutations and one splicing mutation, have been reported so far (Figure 1H). Our study reported the first nonsense mutation that was linked with cardiovascular diseases, located in kinase activity domain. This mutation results in the early appearance of stop codon which is anticipated to produce truncated protein lacking partial protein kinase domain along with the C‐terminus. For all we know, a premature termination codon (PTC) may result in loss of function (LOF) through NMD.^23^, ^24^ NMD refers to rapid degradation of mRNAs in transcripts harboring a PTC existing in all eukaryotic cells, which prevents the synthesis of truncated and potentially toxic proteins. Mutations that lead to NMD can cause a pathological state owing to marked reductions in specific gene expression, predispose patients to some disorders. In our research, the decreased expression of *TNNI3K* mRNA caused by the mutation may lead to CCD through haploinsufficiency.

Nonsense mutation has larger effects in protein function in comparison with missense mutation. Thus, it is anticipated to have more serious phenotype than missense mutation correspondingly. The clinical presentation of cases with *TNNI3K* mutation has been summarized in Table 3. Conduction abnormalities of *TNNI3K* mutation carriers in our case seem to be more serious than previously reported cases based on the fact that all of them had PMs implanted. Cardiac structures were all normal in them except for II‐5 and II‐7 whose heart was enlarged in follow‐up period. Based on the fact that II‐5 and II‐7 are old highly pacing‐dependent male patients with over 30 years' history of PM implantation, we hold the opinion that cardiac enlargement in them was PM‐related and remotely connected with the mutation.^25^ Considering the oldest patient in this family is 93 and four of the five affected members are over 80 years old, it seems that mutation *TNNI3K* c.1441C > T has little effect on life expectancy with appropriate treatments. However, II‐14 died of heart disease at an early age and over half of previously reported families had a history of sudden cardiac death (SCD), which indicated that *TNNI3K* mutation may have connections with high risk of SCD. Further research needs to be done to assess risk of SCD in *TNNI3K* mutation carriers and draw clear genotype‐phenotype relationships of *TNNI3K*.

**Table 3:** Comparison of clinical presentation of cases with *TNNI3K* mutation

	Family 1	Family 2	Family 3	Family 4	Family 5	Family 6	Our case
Mutation	c.1577G > A	c.1615A > G	c.333 + 2T>C	c.2303G > A	c.2303G > A	c.2303G > A	c.1441C > T
First author	Theis, et al (2014)^16^	Xi, et al (2015)^17^	Fan, et al (2018)^18^	Podliesna, et al (2019)^19^	Podliesna, et al (2019)^19^	Podliesna, et al (2019)^19^	—
Affected individuals	7	6	6	15	6	9	5
Conduction disorders	LAFB, RBBB, AVB	AVB, LAFB, RBBB	SB	Prolonged HV interval	AVB, prolonged HV interval	AVB	AVB, RBBB
Rhythm	SR, PAF, MAT, AFL, AT	SR, JET	SR	SVT, AF, MAT	SVT, AT, MAT	SVT, AT, VT	SR, AT
DCM	3	/	4	3	/	1	1
SCD	1	1	1	2	/	/	/
ICD/PM‐implanted	1^a^	1	/	3	/	3	5

Abbreviations: AT, atrial tachycardia; AVB, atrioventricular block; DCM, dilated cardiomyopathy; JET, junctional ectopic tachycardia; LAFB, left anterior fascicular block; MAT, multifocal atrial tachycardia; PAF, paroxysmal atrial fibrillation; PM, pacemaker; RBBB, right bundle branch block; SB, sinus bradycardia; SCD, sudden cardiac death; SR, sinus rhythm; SVT, supraventricular tachycardia; VT, ventricular tachycardia.

^a^ PM‐implanted because of atrioventricular node ablation.

However, there are several limitations in our research. This study lacks direct evidence in elucidating the underlying pathways between *TNNI3K* and CCD. An animal model harboring specific mutation will be needed to elucidate the underlying signaling pathways.

In conclusion, our study successfully identified the first nonsense mutation in *TNNI3K* (c.1441C > T) that was associated with CCD, paying the way for genetic diagnosis for CCD. Considering the obscure pathophysiological mechanisms and complicated variant spectrum of CCD, we suggest that WES could be applied in potential inherited patients to identify novel mutations and improve our understanding of CCD etiology.

## AUTHOR CONTRIBUTIONS

Q‐M L and JL conceived the design and performed the study. JL and DL analyzed the data. JL wrote the original manuscript. Q‐M L, DL, K‐K W, M‐Z L, NL, C‐Y Z, and X‐L S revised the article. All authors reviewed and approved the final manuscript.

## Supporting information

Figure S1

Table S1

Supplementary Material
