# Supplementary figures and images for: Identification of a nonsense mutation in TNNI3K associated with cardiac conduction disease
Source: J Clin Lab Anal. 2020 Jun 11;34(9):e23418. doi: 10.1002/jcla.23418 (PMC7521241; doi:10.1002/jcla.23418)

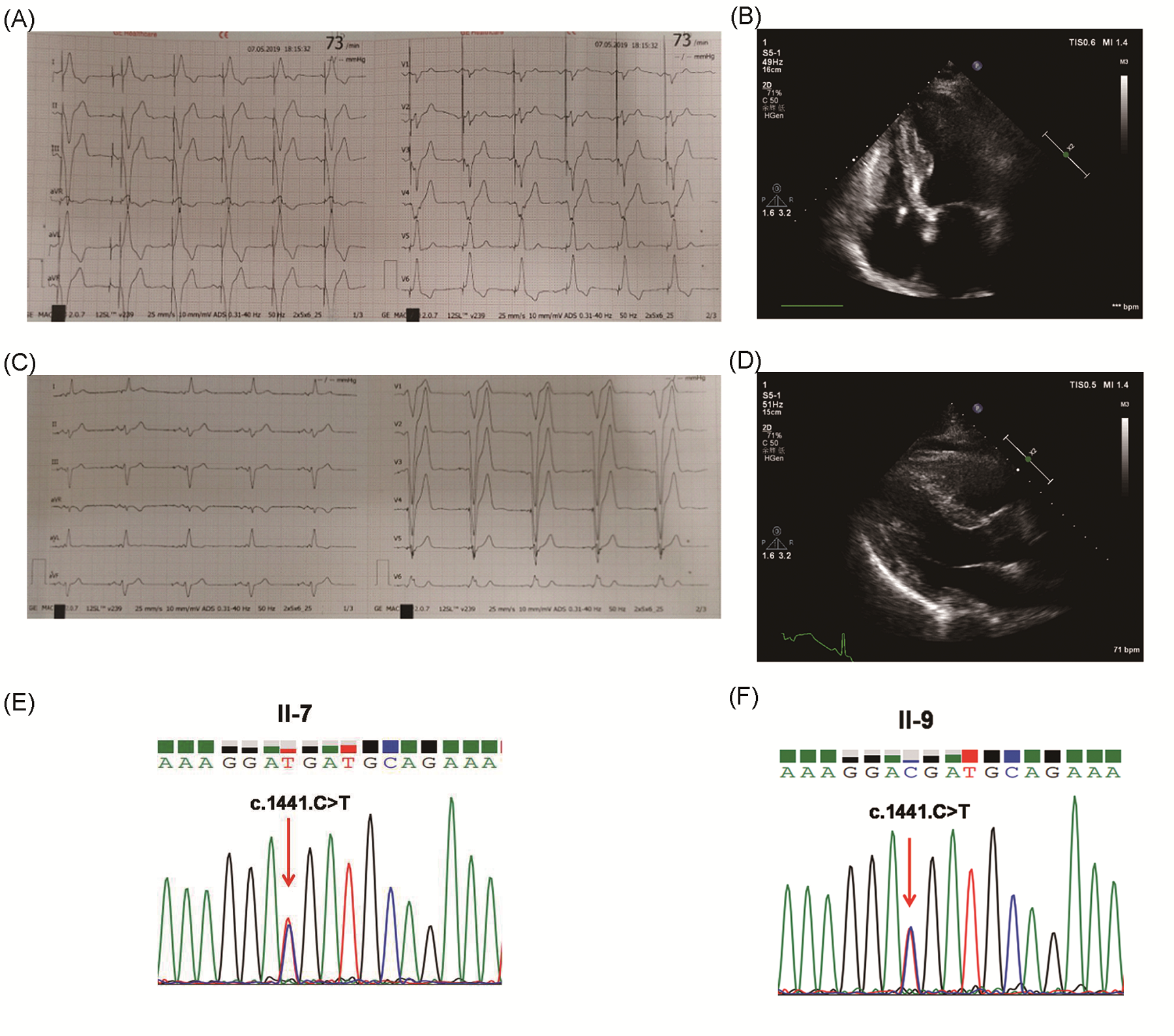

Supplement: Supplementary file 1 — Figure S1 [file JCLA-34-e23418-s001.tif]
