# Supplementary material for: Identification of a nonsense mutation in TNNI3K associated with cardiac conduction disease
Source: J Clin Lab Anal. 2020 Jun 11;34(9):e23418. doi: 10.1002/jcla.23418 (PMC7521241; doi:10.1002/jcla.23418)
Supplement: Supplementary file 2 — Table S1 [file JCLA-34-e23418-s002.docx]

Table S1 Primers Tm and sequences that have been used in co-segregation

| Genes | Forward primer(5’-3’) | Reverse primer(5’-3’) | Production length(bp) | Tm(℃) |
| --- | --- | --- | --- | --- |
| TNNI3K | CCTTCACATTTCCATCTTCAGCT | CTTGGAGCAGTAGGTATTGGC | 362 | 60 |
| RYR2 | TGAGAATGAAACCCTCGACTAC | CATGAGGTCACTGAGCCCA | 1000 | 60 |
| TTN | CATCTGAATCCACTGGGCCT | CCATCACTCTCGGGTCTTGA | 718 | 61 |
| CACNA1C | AGCCAGGCATGAAGAAGGTC | TTCTGTTGTGAAGCCGGAAC | 847 | 61 |
| NUBPL | TGCAAATGTCTTCCAAGCCA | TGAGAAAACTGGTTGGGTGATG | 632 | 62 |
| MYOM1 | ATGTGTCTGCCCCTTATCCC | GGTTCTTGCCTATGTTACTGGG | 598 | 60 |
